# Chromosomal Diversification in *Pseudacanthicus* Species (Loricariidae, Hypostominae) Revealed by Comparative Mapping of Repetitive Sequences

**DOI:** 10.3390/ani12192612

**Published:** 2022-09-29

**Authors:** Kevin Santos da Silva, Augusto César Paes de Souza, Luís Reginaldo Ribeiro Rodrigues, Julio Cesar Pieczarka, Cleusa Yoshiko Nagamachi

**Affiliations:** 1 Laboratório de Citogenética, Centro de Estudos Avançados da Biodiversidade, Instituto de Ciências Biológicas, Universidade Federal do Pará, Belém 66075-750, Brazil; 2 Laboratório de Estudo da Ictiofauna Amazônica, Instituto Federal de Educação, Ciência e Tecnologia do Pará, Abaetetuba 684400-000, Brazil; 3 Laboratório de Genética & Biodiversidade, Instituto de Ciências da Educação, Universidade Federal do Oeste do Pará, Santarém 68040-255, Brazil

**Keywords:** comparative cytogenetics, rDNA, snDNA, repetitive DNA, neotropical ichthyofauna

## Abstract

**Simple Summary:**

The fishes of the Loricariidae family have a huge genetic diversity, mainly involving variations in the number and shape of chromosomes. The recognition of the species genus *Pseudacanthicus* is complex due to the large diversity of forms and limited knowledge of their genetic diversity. In this study, the karyotypes of three *Pseudacanthicus* species were comparatively analyzed using classical and molecular methods. They presented the same diploid number, but with different compositions of repetitive DNA sequences. Such information can be useful for the recognition of distinct species, in addition to providing important insights into the real biodiversity of this important group of Neotropical fish.

**Abstract:**

*Pseudacanthicus* is a genus of Neotropical fish with eight valid species, in addition to numerous lineages not formally identified. It occurs along the Amazon and Tocantins River basins, in Suriname and in the Guiana shield. There are no karyotypic data in the literature for species of this genus. Here, the karyotypes of three *Pseudacanthicus* species (*P. spinosus*, *P. leopardus* and *Pseudacanthicus* sp.) were comparatively analyzed by classical cytogenetics and fluorescence in situ hybridization using 18S and 5S rDNA probes, U2 snDNA and telomeric sequences. The analyzed species presented 52 chromosomes and KF = 18 m + 34 sm. Constitutive heterochromatin occurred in blocks on a few chromosomes. The 18S rDNA occurred in a single pair; interestingly, *P. leopardus* presented only one locus of this sequence in its diploid genome. The 5S rDNA sequence occurred in only one pair in *P. leopardus*, and in multiple sites in *Pseudacanthicus* sp. and *P. spinosus*. The snDNA U2 occurred in only one pair in all analyzed species. Telomeric sequences did not show interstitial sites. Although *Pseudacanthicus* species share the same 2n and KF, repetitive sequence analysis revealed karyotypic diversity among these species. The occurrence of DNA double-strand breaks related to fragile sites, unequal crossing over and transpositions is proposed as the mechanism of karyotypic diversification, suggesting that the conservation of the karyotypic macrostructure is only apparent in this group of fish.

## 1. Introduction

The Loricariidae family represents one of the most diverse groups of Neotropical fish [1]. These fish occur in Central and South America, from southeastern Costa Rica to northeastern Argentina [2,3]. Currently, six subfamilies are recognized for Loricariidae: Lithogeninae, Delturinae, Hypoptopomatinae, Neoplecostominae, Loricariinae and Hypostominae; the latter one is the most diverse, widely distributed and systematically complex [1,4]. Lujan et al. [4] retrieved a phylogeny for Hypostominae based on molecular data (mitochondrial and nuclear genes), recognizing it as a monophyletic group organized into nine clades and tribes: tribe Ancistrini, tribe Hypostomini, clade *Chaetostoma*, clade *Pseudancistrus*, clade *’Pseudancistrus’*, clade *Lithoxus*, clade *Hemiancistrus*, clade *Peckoltia* and clade *Acanthicus*.

*Pseudacanthicus* Bleeker, 1862 (Hypostominae, clade *Acanthicus*) is a genus of Neotropical fish distributed along the Amazon and Tocantins River basins in Suriname and the Guiana shield [1,5,6]. Currently, this genus has eight valid species, in addition to numerous lineages not formally identified [1,5,6,7]. Representatives of *Pseudacanthicus* have great taxonomic complexity, being generically recognized through ambiguous morphological characters, such as external coloration patterns, which justifies the large number of unidentified lineages formally associated with this genus [5,6,7]. It is worth noting that *Pseudacanthicus* species exhibit body shapes and coloration patterns that stand out in the practice of aquarism, making the species of this genus a target of intense commercialization in the national and international ornamental fish market [5,7].

Karyotypic analyses have been established as an important approach for describing the biodiversity of fish and other systematically complex vertebrate groups [8,9,10]. Comparative analyses have shown extensive numerical and structural chromosomal diversity among species of the Loricariidae family, with 2n ranging from 34 to 96 chromosomes [11,12]. In the subfamily Hypostominae, a large variation in 2n has been observed, mainly between species of the genera *Ancistrus* and *Hypostomus* [12,13]; on the other hand, representatives of other Hypostominae lineages with known karyotypes do not show any variation in 2n [10,13,14]. Repetitive DNA sequences correspond to the largest portion of eukaryotic genomes, and may be organized in tandem (e.g., ribosomal genes) or dispersed (e.g., transposable elements—TEs) [15]. The ribosomal genes (rDNA) represent two distinct gene families: 45S ribosomal DNA (5.8S + 18S + 28S genes) and 5S ribosomal DNA [16]. The genes encoding small nuclear RNAs (snDNA) represent another gene family organized in in tandem repeats constituted by the genes *U1*, *U2*, *U4*, *U5* and *U6*, which express non-coding RNAs involved in the maturation of messenger RNA in eukaryotes [17]. These groups of repetitive sequences have been useful as markers in comparative cytogenetic analyses, improving the understanding of mechanisms of karyotypic diversification in different groups of fish [9,10,18,19,20,21]. Furthermore, these analyses have been efficient in evidencing mechanisms of chromosomal differentiation between species whose karyotypic constitution appears to be homogeneous, representing an important approach to the study of karyotypic diversification in these groups of organisms, as observed in different lineages of the subfamily Hypostominae [10,22,23].

In this paper, the karyotypes of three species of the genus *Pseudacanthicus* were analyzed using chromosome banding techniques and a comparative in situ mapping of repetitive sequences. The mechanisms of chromosomal differentiation involving repetitive DNA sequences are discussed.

## 2. Materials and Methods

### 2.1. Samples

Samples from three *Pseudacanthicus* species (*Pseudacanthicus* sp., *P. leopardus* and *P. spinosus*) were analyzed. The specimens of *P. spinosus* were collected in the Tocantins-Araguaia River basin, Brazil (S01°45′18.2″/W49°00′38.8″) in November, 2018. To carry out comparative analyses, specimens of *P. leopardus* and *Pseudacanthicus* sp. acquired in aquarium stores in the city of Belém, Brazil, were analyzed. Details on the origin, number of specimens, sex and deposit in zoological collections are described in Table 1. The taxonomic identity of the species analyzed in this study was confirmed by DNA barcoding. Details on isolation, sequencing and analysis to identify the species analyzed by DNA barcode are described in the supplementary data section (Supplementary Table S1). The Instituto Chico Mendes da Conservação da Biodiversidade—ICMBio authorized the collection of samples in nature (license number 13248). The Cytogenetics Laboratory of the Centro de Estudos Avançados da Biodiversidade—CEABIO of UFPA has a license granted by the Ministry of the Environment for transport (number 19/2003) and use of animals in research (number 52/2003). The Ethics Committee of the Universidade Federal do Pará approved this research (permission 68/2015). The analyzed specimens were deposited in the ichthyology collection of the Centro de Estudos Avançados da Biodiversidade, Universidade Federal do Pará, Brazil.

### 2.2. Chromosomal Analysis 

The animals were anesthetized with eugenol and euthanized for extraction of metaphasic chromosomes from kidney cells after treatment with colchicine (0.025%) in vivo, as described in literature [24]. Chromosomes were analyzed by conventional staining with 5% Giemsa diluted in phosphate buffer (pH 6.8), C-banding [25] and fluorescent in situ hybridization [26].

### 2.3. Probes Labeling and Fluorescence In Situ Hybridization

The 18S rDNA, 5S rDNA, U2 snDNA and telomeric sequences probes were used in fluorescence in situ hybridization (FISH) experiments. Genomic DNA was extracted using the Wizard Genomic DNA Purification Kit (Promega, Madison, WI, USA) according to the manufacturer’s instructions. The 5S rDNA and U2 snDNA probes were isolated by polymerase chain reaction (PCR) from *P. spinosus* genomic DNA using the primer set described [27,28], respectively. The 18S rDNA probes (~1400 bp follow-up) were obtained from the genome of the fish *Prochilodus argenteus* [29]. Telomeric probes were obtained according to literature [30]. The probes were labeled with biotin or digoxigenin using a nick translation kit. FISH experiments were performed [26], with modifications, under the following stringency conditions: 2.5 ng/μL of each probe, 50% formamide, 2 × SSC, 10% dextan sulfate and hybridization at 42 °C for 16 h. Hybridization signals were detected with FITC (green) and Cy3 (red), and chromosomes were counterstained with 4′-6-diamidino-2-phenylindole (DAPI; blue).

### 2.4. Image Capture and Analysis

Approximately 30 metaphases were analyzed for each sample to determine diploid number (2n), fundamental number (FN) and karyotypic formula (KF) using chromosome banding. To verify the results of the FISH experiments, close to 30 metaphases were analyzed. Conventional cytogenetic techniques images (Giemsa staining and C-banding) were obtained using an Olympus BX41 microscope (brightfield) with the aid of a CCD 1300QDS digital camera and analyzed using GenASIs software version 7.2.7.34276 (Applied Spectral Imaging, Carlsbad, CA, USA). FISH images were obtained using a Nikon H500S photomicroscope equipped with Nis-Element Software version 4.0 (Melville, NY, USA). Chromosomes were organized following the chromosomal morphology classification criteria proposed [31]. Karyotypes were organized using the Adobe Photoshop CS6 program.

## 3. Results

### 3.1. Classical Cytogenetics

**Figure 1:**
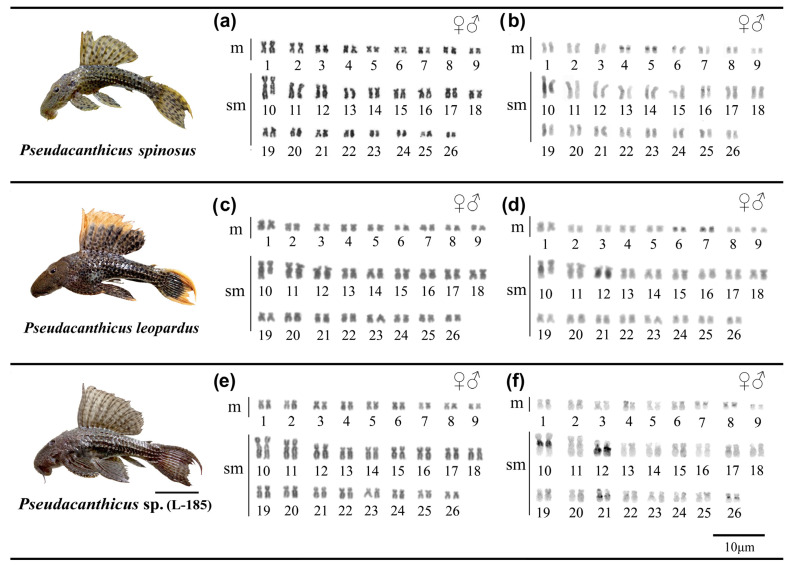
Karyotypes of *Pseudacanthicus* species. In (**a**,**b**) karyotypes *of P. spinosus*; in (**c**,**d**) karyotypes of *P. leopardus*; and in (e,**f**) karyotypes of *Pseudacanthicus* sp. after conventional staining with Giemsa and C-banding, respectively. The external coloration patterns of the analyzed *Pseudacanthicus* species are shown on the side, scale bar: 5 cm. Photos by Kevin Santos da Silva.

The *Pseudacanthicus* species analyzed show karyotypes with 2n = 52 chromosomes (KF = 18 m + 34 sm) and FN = 104. None of the analyzed species presented heteromorphic sex chromosomes (Figure 1a,c,e; Table 2). In *P. spinosus*, constitutive heterochromatin (CH) occurred in the centromeric region of pairs 4, 5 and 16, and proximal to the long arm (q) of pair 10, with size heteromorphism between the homologues of this pair (Figure 1b); in *P. leopardus*, CH occurred in the centromeric region of pairs 6 and 7, interstitial of pair 12q and proximal of only one of the homologues of pair 10q, with heteromorphism in size between the homologs (Figure 1d); and, in *Pseudacanthicus* sp., CH occurred in the centromeric regions of pairs 7, 8 and 26, interstitial in pair 21q and proximal in pairs 10q and 12q (Figure 1f).

### 3.2. Molecular Cytogenetics

**Figure 2:**
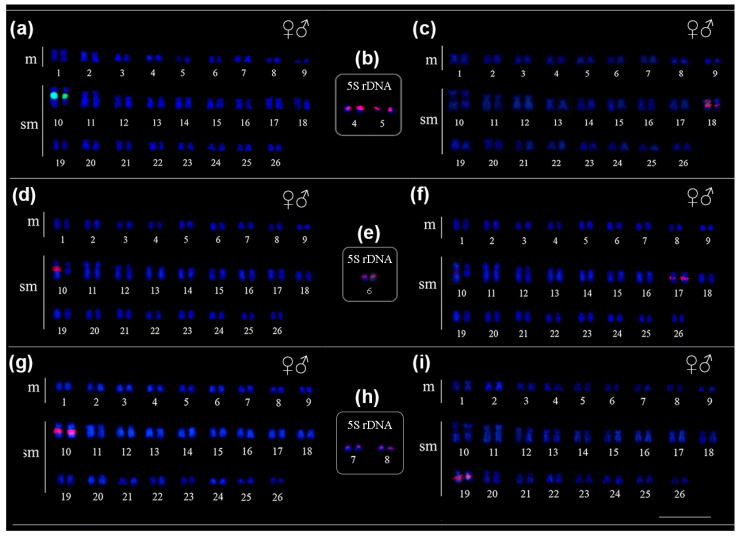
Fluorescence in situ hybridization indicating the physical location of rDNA and snDNA sequences in the karyotypes of *Pseudacanthicus* species. In (**a**) 18S rDNA, (**b**) 5S rDNA and (**c**) U2 snDNA in *P. spinosus*; in (**d**) 18S rDNA, (**e**) 5S rDNA and (**f**) U2 snDNA in *P. leopardus*; in (**g**) 18S rDNA, (**h**) 5S rDNA and (**i**) U2 snDNA in *Pseudacanthicus* sp. Hybridization signals were detected with FITC (green) and Cy3 (red), and chromosomes were counterstained with DAPI (blue).

**Figure 3:**
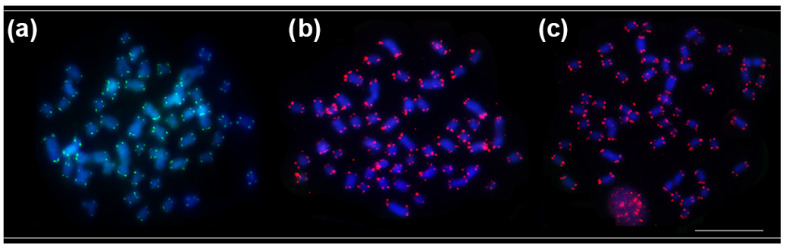
Fluorescence in situ hybridization indicating the physical location of telomeric sequences in the karyotypes of *P. seudacanthicus* species. In (**a**) *P. spinosus*, in (**b**) *P. leopardus*, and in (**c**) *Pseudacanthicus* sp. Hybridization signals were detected with FITC (green) and Cy3 (red), chromosomes were counterstained with DAPI (blue).

The in situ location of repetitive sequences in *Pseudacanthicus* is described in Table 2. In *Pseudacanthicus* sp. and *P. spinosus*, the 18S rDNA sequence was located in the proximal region of the 10q submetacentric pair (Figure 2a,g); in *P. leopardus*, this sequence occurred in only one of the homologues, colocalized with the CH and associated with a marked heteromorphism in size between the homologues of the 10q chromosome pair (Figure 2d). The 5S rDNA occurred at multiple sites in the pericentromeric region of pairs 4 and 5 in *P. spinosus* (Figure 2b) and pairs 7 and 8 in *Pseudacanthicus* sp. (Figure 2e); in *P. leopardus*, this sequence occurred in the pericentromeric region of pair 6 (Figure 2h). The U2 snDNA occurred in the interstitial region of only one pair of submetacentric chromosomes in all species: in pair 18q in *P. spinosus*, in pair 17q in *P. leopardus* and in pair 19q in *Pseudacanthicus* sp. (Figure 2c,f,i, respectively). The mapping of telomeric sequences did not show interstitial telomeric sites (ITS) in any of the analyzed karyotypes (Figure 3a–c).

## 4. Discussion

In Loricariidae, there is evidence of extensive karyotype diversity, with an emphasis on the variation in 2n (ranging from 2n = 34 to 96), with a supposed ancestral karyotype of 2n = 54 [11,32]. In the subfamily Hypostominae, many of the variations in diploid number are observed in species of the genera *Ancistrus*, tribe Ancistrini (2n = 34 to 54) [33] and *Hypostomus*, tribe Hypostomini (2n = 64 to 84) [34,35]. However, the karyotypes known so far for species of the clades *Acanthicus*, *Hemiancistrus* and *Peckoltia* and basal lineages of the tribe Hypostomini (e.g., genus *Pterygoplichthys*) do not show any variation in diploid number (2n = 52) [9,10,13]. According to Bueno et al. [13], 2n = 52 may represent an ancestral character for these lineages in Hypostominae. Considering the hypothesis that 2n = 54 represents an ancestral character for the family Loricariidae, the reduction to 2n = 52 can be explained by the occurrence of Robertsonian rearrangements without the maintenance of ITS in the common ancestor of the species of the clades *Acanthicus*, *Hemiancistrus* and *Peckoltia* and tribe Hypostomini, as suggested by telomeric sequence mapping data in different species of these groups (Ref. [10] and present study).

In the subfamily Hypostominae, most of the species share the diploid number; however, an extensive variation in karyotypic morphology is observed, suggesting the occurrence of rearrangements such as inversions and translocations [10,12,36]. The involvement of repetitive sequences in chromosomal differentiation has been demonstrated in several groups of vertebrates, including mammals [37,38], amphibians [23] and other fish species of the subfamily Hypostominae [9,10,20]. In *Pseudacanthicus*, variations in the karyotypic formula were not evident; however, the comparative analysis of the distribution of CH blocks, as well as of the rDNA and snDNA genes, suggested the occurrence of microstructural differentiation events among the analyzed karyotypes, including transpositions, CH amplification/deletions and unequal crossing-over. Furthermore, the distribution of these markers showed specific characteristics for the karyotypes of the *Pseudacanthicus* species analyzed here, indicating the relevance of these analyses for understanding the karyotypic differentiation and recognition of these systematically complex lineages.

Comparative analyses involving different classes of repetitive DNA have been useful in demonstrating chromosomal diversity among different groups of organisms, including those with little or no variation in karyotypic macrostructure (Refs. [10,36] and present study). The evolution of rDNA genes represents a very informative aspect of chromosomal diversity in fish [39,40]. In Loricariidae, the syntenic location of the 18S/5S rDNA genes represents a plesiomorphic character [12]; however, extensive variation in the position and number of sites are observed for these repetitive sequences in the karyotypes of most of the analyzed species [13,14]. In *Pseudacanthicus*, the 18S/5S rDNA genes showed a non-synthetic distribution in all analyzed species, similar to that observed in *Megalancistrus parananus*, another species of the *Acanthicus* clade [13]. This synteny break may be associated with the existence of evolutionary breakpoints adjacent or internal to the rDNA genes, which may facilitate the occurrence of rearrangements involving these genes in Loricariidae, as previously proposed for other representatives of this family [10,13,20].

The 5S rDNA represents one of the most dynamic repetitive sequences in fish genomes, being associated with several differentiating mechanisms and chromosomal rearrangements [20,40]. In Loricariidae, the involvement of these sequences in chromosome breakage and rearrangement events has been proposed to justify the presence of this sequence at chromosomal fusion points, as well as the occurrence of multiple sites in the karyotypes of different species [20]. Alternatively, the dispersion of these sequences may be related to transposition events [15,41,42]. Different families of TEs are associated with 5S rDNA genes, which may play a central role in the formation of additional sites in these sequences [40]. In *Pseudacanthicus*, both the 18S and 5S rDNA genes were associated with CH regions, in agreement with what was observed in the karyotypes of other fish groups [15,41,42]. Centromeric heterochromatin regions are characterized by the presence of several classes of highly repetitive DNA, including TEs, and represent chromosomal regions that are more susceptible to breakage events, non-homologous recombination and transpositions [41,42]. The occurrence of transpositions and duplications facilitated by associated TEs may represent plausible mechanisms that explain the dispersion of 5S rDNA sequences in the karyotypes of *Pseudacanthicus* sp. and *P. spinosus*, since the appearance of multiple sites in this sequence does not seem to be related to structural rearrangements in the genomes of these species [40,43]. Future analyses will be important to test the hypothesis of the association between TEs and rDNA sequences in *Pseudacanthicus*.

The occurrence of 18S rDNA in the interstitial region of only one pair of chromosomes is considered a plesiomorphic condition in Loricariidae [32]. In *Pseudacanthicus*, the 18S rDNA occurred in only one pair of morphologically similar chromosomes, suggesting the maintenance of the ancestral condition. However, different degrees of size heteromorphism were observed between the homologues of the 18S-rDNA-bearing chromosome pair among *Pseudacanthicus* species, being more pronounced in *P. leopardus*, where only one locus of this repetitive sequence was observed in the diploid genome. Unequal crossing-over events are commonly related to the occurrence of size heteromorphisms between regions rich in repetitive DNA in fish, including regions carrying rDNA genes [44], which may explain the size heteromorphism observed between homologs of the 18S rDNA-bearing pair (par 10) in *Pseudacanthicus* sp. and *P. spinosus*; however, this does not seem to be the case for *P. leopardus*. In this species, the condition observed for 18S rDNA suggests the occurrence of double-stranded DNA breaks and the deletion of the region carrying this repetitive sequence in one of the homologues of pair 10.

Fragile sites for chromosomal breaks associated with rDNA sequences is a phenomenon documented in the genomes of different organisms, including humans [37], rodents [38], plants [45] and fish [20], being associated with the occurrence of chromosomal rearrangements in these organisms. There is evidence that chromosomal breaks associated with 45S rDNA sites can occur independently at any of the loci, as observed in *Loliun* spp. [45]. We suggest that the occurrence of breaks and deletion may explain the condition observed for 18S rDNA in *P. leopardus*, since the occurrence of unequal crossing-over or CH amplification/deletion events do not seem to fully explain the marked size heteromorphism, as well as the different DNA contents observed between the homologues of the 18S-rDNA-bearing pair in this species. Future analyses will be important to verify if this characteristic represents an autapomorphy or just a population variation in *P. leopardus*.

Analyses involving snDNA genes have shown great chromosomal diversity in different groups of organisms [18,21,46,47]; however, these genes represent one of the least explored gene families in chromosomal studies among fish species [21]. These repetitive sequences exhibit intense diversity in terms of distribution pattern, which may be dispersed, in blocks or even dispersed in small blocks [10,18,21,48,49]. In *Pseudacanthicus*, the U2 snDNA occurred in single sites located on morphologically similar chromosomes, indicating a similar chromosomal organization among the species analyzed here. This hypothesis is supported when we compare the distribution of these genes among the karyotypes of species from different groups, such as species of the genera *Astyanax* [50] and *Pimelodus*, another group of Siluriformes [51]. On the other hand, snDNA gene mapping has shown variations in the number of sites in other loricariids, as observed in *Peckoltia* species (Hypostominae, *Peckoltia* clade), where single and multiple U1 snDNA sites occur between different species [10]. Thus, although the in situ locations of these sequences may indicate stability at the chromosomal level, chromosomal and molecular diversity has been observed for this group for repetitive sequences in the genomes of different fish species [18,52,53]. Therefore, analyzing the in situ locations of other snDNA genes, as well as the molecular diversity of this gene family in the genomes of *Pseudacanthicus* species, may reveal important mechanisms of chromosomal diversification involving this group of repetitive sequences in this group of fish.

## 5. Conclusions

In this paper, the karyotypes of species of the genus *Pseudacanthicus* are described for the first time. Our data show that, although those species share a similar 2n and karyotype morphology, chromosomal reorganization events involving regions rich in repetitive sequences may explain the variations observed in the location of the CH, and rDNA and snDNA sequences may have occurred throughout the karyotypic evolution of these species; in addition, the data suggest that the conservation of the observed macrostructure of the karyotype is only apparent, indicating that repetitive sequence analysis represents an interesting approach for the distinguishing and recognition of lineages belonging to the genus *Pseudacanthicus*.

## Figures and Tables

**Table 1:** Samples of *Pseudacanthicus* species analyzed in this study.

Species	L-Number Code	Sex	Voucher *	Rivers	Hydrographic Basin
*Pseudacanthicus spinosus*	L-160	6♂/2♀	*P4212*	Tocantins river	Tocantins-Araguaia basin
*Pseudacanthicus leopardus*	L-114	1♂/1♀	*P4252*	Xingu river	Amazon basin
*Pseudacanthicus* sp.	L-185	1♂/1♀	*P4258*	Xingu river	Amazon basin

Legends: (*) Ichthyology collection in the Centro de Estudos avancados da biodiversidade, CEABIO, UFPa, Brazil.

**Table 2:** Chromosomal data of the *Pseudacanthicus* species analyzed in this study.

Species	Karyotypes			Molecular Cytogenetics			
	2n	NF	KF	rDNA 18S	rDNA 5S	snDNA U2	Tel.
***Pseudacanthicus spinosus***	52	104	18 m + 34 sm	10q	4, 5	18q	distal
***Pseudacanthicus leopardus***	52	104	18 m + 34 sm	homologue 10q	6	17q	distal
***Pseudacanthicus*** **sp.**	52	104	18 m + 34 sm	10q	7, 8	19q	distal

Legends: 2n—diploid number, FN—fundamental number, KF—karyotypic formula, rDNA—*ribosomal* genes, snDNA—*small nuclear* RNA genes.

## Data Availability

Not applicable.

## Supplementary Materials

The following supporting information can be downloaded at: https://www.mdpi.com/article/10.3390/ani12192612/s1, Data Supplementary–DNA barcoding methods and DNA barcoding sequences obtained for *Pseudacanthicus* species analyzed in this study, Supplementary Table S1: Taxonomic identification of *Pseudacanthicus* specimens based on DNA barcode (COI sequence) similarity through ID engine tool (www.boldsystems.org, 13 May 2021).
