# Supplementary material for: Chromosomal Diversification in Pseudacanthicus Species (Loricariidae, Hypostominae) Revealed by Comparative Mapping of Repetitive Sequences
Source: Animals (Basel). 2022 Sep 29;12(19):2612. doi: 10.3390/ani12192612 (PMC9558496; doi:10.3390/ani12192612)
Supplement: Supplementary file 1 [file animals-12-02612-s001.zip › animals-1901887-SI.pdf]

Title: Chromosomal diversification in *Pseudacanthicus* species (Loricariidae, Hypostominae) revealed by comparative mapping of repetitive sequences

Authors: Kevin Santos da Silva, Augusto César Paes de Souza, Luís Reginaldo Ribeiro Rodrigues, Julio Cesar Pieczarka, Cleusa Yoshiko Nagamachi.

Data Supplemental file:

DNA barcoding methods

DNA extraction was processed with a column based commercial kit PureLink Genomic DNA Kit (ThermoFisher Scientific) and the amounts were evaluated in a 1% agarose gel stained with Gelred (Biotium). DNA barcode sequence of the *Cytochrome c Oxidase subunit I* (COI) mitochondrial gene were amplified using the universal primers Fish F1 and Fish R1. The reactions were assembled in 25 µL, containing 15 µL sterile H_2O, 2.8 µL dNTP mix (1.25 mM), 2.5 µL buffer 10X (200 mM Tris-HCl (pH = 8,4) + 500 mM KCl), 2.5 µL $MgCl_2$ (50 mM), 0.5 µL of each primer (5µM), 0.2 µL Taq DNA polymerase (5U/µL) and 1 µL of genomic DNA (around 100ng). The cycling profile followed as 95°C/2min, 35 cycles of 94°C/30sec, 54°C/30sec and 72°C/1min, and a final step of 72°C/10min. in a Pxe 0.2 thermocycler (ThermoFisher Scientific). We evaluated the amplicons with a 1% agarose gel stained with Gelred and purified the PCR products with PEG8000. DNA barcode sequences were generated in an ABI 3500 genetic analyzer (Applied Biosystems) using the chemistry Big Dye Terminator V.3 Cycle Sequencing kit (Applied Biosystems, Waltham, Massachusetts, USA).

Molecular taxonomic identification

The chromatograms of COI sequences were inspected for base calling quality and converted to fasta file format, using the programs FinchTV v1.4.0 and BioEdit v. 7.2.5. The DNA barcodes (fasta file) were pasted in the ID Engine interface (www.boldsystems.org) and processed for taxonomic identification based on a percentage of sequence similarity against a DNA barcode library currently constituted with 4.7 million of COI sequences, representative of 241,692 species. We assumed a cutoff of 97% similarity for correct species identity, in order to recover the best sequence match with DNA barcodes linked to formally curated nominal taxa.

DNA barcode sequences obtained for *Pseudacanthicus* species analyzed in this study:

Pseudacanthicus spinosus
>P4212
TTCTTRGGGAYYGAGTGSCCGAATCTGGCATGGTTGGCCAGTTCCTCASTCTTTTTTTCGA
GCTGAGCTRAGCAACCCGGTTCTMTACTARGTGATGACCAAATTTATAATGTCATCGTTACT
GCACATGCTTTTGTAATGATTTTCTTTATAGTAATACCGATTATGATTGGAGGCTTTGGAAAT
TGACTAGTACCACTAATAATTGGAGCACCTGACATAGCCTTTCCACGAATAAATAATATAAG
CTTCTGATTACTTCCACCTTCATTCCTTCTACTACTAGCCTCTTCAGGAGTTGAAGCGGGAG
CAGGGACAGGTTGGACTGTATATCCTCCACTCGCTGGAAATTTAGCTCACGCAGGAGCTTC
AGTAGACCTTACTATCTTTTCACTTCATCTAGCTGGTGTCTCCTCAATTCTTGGAGCAATTAA
CTTTATCACTACAATCATTAATATAAAACCCCCAGCTATTTCACAATACCAAACCCCTTTATT
TGTATGAGCCTTACTTATTACAGCTGTCCTACTCCTACTTTCACTACCAGTTCTAGCTGCTG
GAATCACAATGTTATTAACAGACCGAAACCTGAATACTACCTTCTTCGACCCTGCAGGAGG
CGGGGACCCAATCCTTTACCAACACTTATTCTGATTCTTTGGCCACCCRARAARTMGGTWS
AACCAAC

Pseudacanthicus leopardus
>P4252
TTTWATAGCTGCTTGGTGCTTGAGCCGGATGGTTGGCACAGCCCTCAGCCTTTTAATTCGA
GCTGAGCTAAGCCAACCCGGTTCTCTACTAGGTGATGACCAAATTTATAATGTCATCGTTAC
TGCACATGCCTTTGTAATGATTTTCTTTATAGTAATACCAATTATGATTGGAGGCTTTGGAAA
CTGACTAGTACCACTAATAATTGGAGCACCTGACATAGCCTTTCCACGAATAAATAATATAA
GCTTCTGACTACTTCCACCTTCATTCCTTTTACTACTAGCCTCTTCAGGAGTTGAGGCGGGA
GCAGGGACAGGTTGGACTGTATATCCTCCACTCGCTGGAAATTTAGCCCACGCAGGAGCT
TCAGTAGACCTTACTATCTTTTCACTTCATCTAGCTGGTGTCTCCTCAATTCTTGGAGCAATT
AACTTTATCACTACAATCATTAATATAAAACCCCCAGCTATTTCACAATACCAAACCCCTTTA
TTTGTATGAGCCTTACTTATTACAGCTGTACTACTCCTACTTTCACTGCCTGTTCTAGCTGCT
GGAATTACAATGTTACTAACAGACCGAAACCTAAATACTACCTTCTTCGACCCTGCAGGAG
GCGGGGACCCAATCCTTTACCAACACTTATTCTGATTCTTTGGCCACCCRGAAAGTCTGTA
AAAACCC

Pseudacanthicus sp.
>P4258
TTMMTTAGTGTTTGGTGCCTGAGCTGGATGGTTGGCACAGCCCTCAGCCTTTTAATTCGAG
CTGAGCTAAGCCAACCCGGTTCTCTATTAGGTGATGACCAAATTTATAATGTCATCGTTACT
GCACATGCTTTTGTAATGATTTTCTTTATAGTAATACCAATTATGATTGGAGGCTTTGGAAAT
TGACTAGTACCACTAATAATTGGAGCACCTGACATAGCCTTTCCACGAATAAATAATATAAG
CTTCTGATTACTTCCACCTTCATTCCTTCTACTACTAGCCTCTTCAGGAATTGAAGCGGGAG
CAGGGACAGGTTGGACTGTATATCCTCCACTCGCTGGAAATTTAGCTCACGCAGGAGCTTC
AGTAGACCTTACTATCTTTTCACTTCATCTAGCTGGTGTCTCCTCAATTCTTGGAGCAATTAA
CTTTATCACTACAATCATTAATATAAAACCCCCAGCTATTTCACAATACCAAACCCCTTTATT
TGTATGAGCCTTACTTATTACRGCTGTCCTACTCCTACTTTCACTACCAGTTCTARCTGCTG
GAATCACARTGTTRKTAACAGACCGAAASCTGAATACTRCCTTCKKCGRCCCTRCASGAGG
CGGGGACSSAATCCTTTACCAACACTTRKKMRARAWWCTTWRARMCACSCAGRRAAGKCT
GTK

Note*: These sequences are showed in forward direction without edition or manipulation.*

Table S1: Taxonomic identification of *Pseudacanthicus* specimens based on DNA barcode (COI sequence) similarity through ID engine tool (www.boldsystems.org).

Voucher*	Field number	Species	Molecular ID (COI sequence)	% similarity
P4212	QDB_16	*Pseudacanthicus spinosus*	*Pseudacanthicus spinosus*	99.17
	QDB_17	*Pseudacanthicus spinosus*	*Pseudacanthicus spinosus*	97.05
	QDB_18	*Pseudacanthicus spinosus*	not evaluated	-
	QDB_19	*Pseudacanthicus spinosus*	*Pseudacanthicus spinosus*	98.00
	QDB_20	*Pseudacanthicus spinosus*	*Pseudacanthicus spinosus*	99.84
	QDB_21	*Pseudacanthicus spinosus*	*Pseudacanthicus spinosus*	99.83
	QDB_22	*Pseudacanthicus spinosus*	*Pseudacanthicus spinosus*	99.84
	QDB_23	*Pseudacanthicus spinosus*	*Pseudacanthicus spinosus*	98.05
P4252	IBA_1	*Pseudacanthicus leopardus*	*Pseudacanthicus leopardus*	99.83
	IBA_2	*Pseudacanthicus leopardus*	*Pseudacanthicus leopardus*	100
P4258	IBA_3	*Pseudacanthicus* sp.	*Pseudacanthicus* sp.; *P. spinosus*	99.68; 99.67
	IBA_4	*Pseudacanthicus* sp.	not evaluated	-

Legends: (*) Coleção de Ictiologia do Centro de Estudos Avançados da Biodiversidade - CEABIO, UFPA, Brazil.
